# ParaDockS - an open-source framework for molecular docking: implementation of target-class-specific scoring methods

**DOI:** 10.1186/1758-2946-5-S1-P11

**Published:** 2013-03-22

**Authors:** Michael Scharfe, Martin Pippel, Wolfgang Sippl

**Affiliations:** 1 Institute of Pharmacy, University Halle-Wittenberg, Halle, 06120, Germany

## 

Accurate scoring of protein-ligand interactions in molecular docking and virtual screening is still challenging. Despite great efforts, the performance of existing scoring functions strongly depends on the target structure under investigation. Recent developments in the direction of target-class-specific scoring methods and machine-learning-based classification models reveals a significant improvement in binding mode and activity prediction [1].

However, there is currently no open-source framework available that combines novel scoring techniques with molecular docking algorithms to make this simply applicable for virtual screening. Therefore, the aim of this work is the implementation and validation of target-biased scoring methods within the open-source docking framework ParaDockS [2]. ParaDockS includes algorithms for protein-ligand docking and is organized that every newly developed scoring function can be immediately implemented. Furthermore, interaction-based classifier, trained on a target-specific knowledge base can be used in a post-docking filter step.

In general, we focus on knowledge-based scoring functions based on well established statistical potentials. Recently it was shown, that atom-pair potentials are also useful for training machine-learning models based on support vector machines or random forest approaches [1]. Such methods circumvent a particular functional form for the scoring function and thereby implicitly capture binding contributions that are hard to model explicitly.

In a first validation study, we applied newly developed target-specific potentials on different kinase data sets and we found that they outperform scoring functions that are not tailored to this target class. Due to the open-source and modular architecture of ParaDockS further scoring funtions can be easily implemented and immediately used for docking and virtual screening.

The whole ParaDockS suite is distributed under the terms of the GNU General Public License Version 2 and is free to download, to reuse, to modify and to redistribute any file of the source code.

## References

[B1] Li Target-Specific Support Vector Machine Scoring in Structure-Based Virtual Screening: Computational Validation, In Vitro Testing in Kinases, and Effects on Lung Cancer Cell Proliferation J Chem Inf Model 2011 51 755 759 10.1021/ci100490w 21438548 PMC3092157

[B2] Meier, Pippel, Brandt, Sippl, Baldauf ParaDockS - A framework for Molecular Docking with Population-Based Metaheuristics J Chem Inf Model 2010 50 879 889 10.1021/ci900467x 20415499

